# Dealing with bereaved children: a case study

**DOI:** 10.29045/14784726.2021.3.5.4.54

**Published:** 2021-03-01

**Authors:** Ben McGachy

**Affiliations:** South Western Ambulance Service NHS Foundation Trust

**Keywords:** childhood bereavement, childhood grief, parental loss, traumatic grief

## Abstract

**Introduction:**

A personal reflection on managing bereaved children (BC) following unexpected death of a parent.

**Questions:**

What evidence is available to assist ambulance clinicians when supporting BC?

**Methods:**

A literature search on BC in pre-hospital environments was undertaken.

**Results:**

Paucity of literature necessitated search expansion beyond pre-hospital/ambulance focus, and use of supplementary sources of credible information from registered bereavement charities and help groups.

**Conclusion:**

More research is needed to better support this vulnerable, unique demographic. It is hoped that this article will encourage further discussion and research into this topic.

## Introduction

Approximately 4–5% of UK children suffer parent loss before their 18th birthday, which is associated with adverse health outcomes throughout life (Ellis et al., 2013). Children grieve as deeply as adults (Child Bereavement UK, 2018), yet little is known about the differing course of grief in bereaved children (BC), meaning they are often forgotten mourners in modern multi-faith and insular societies (Aynsley-Green, 2017).

Misperceptions and poor understanding surround childhood grief. Until relatively recently, whether or not children were even capable of grief was debated and considered by many as unlikely (Bowlby, 1980).

## Case presentation

A 9-year-old girl was at home playing with her father, and witnessed him collapse and turn blue. The child tried to call her mother at work for help, without success. This child then went into the street and, hearing her distress, neighbours called 999. Ambulance and police resources were dispatched to scene and the father was found on the lounge floor in cardiac arrest, dying at scene despite extensive resuscitative efforts, with thromboembolic derangement the suspected cause of death. While the police located the mother, this author cared for the girl for some time, who was verbalising guilt and regret and asking challenging, uncomfortable and difficult questions about her father’s death. The decision was made to honestly and compassionately explain that her father had died, leading to further difficult questions about death, corpses, mortality and the future. While this clinician had limited experience of supporting surviving parents in similar circumstances, supportive deferral to a guiding consent-authorising figure was not possible, and this child was deprived of the love and physical comfort of the surviving parent. In the immediacy of the circumstances, the burden of responsibility and decision-making for managing this child fell solely to this clinician until the mother could get to scene. With clinical knowledge found wanting, intuition and instinct informed decision-making. Having a child of the same age, this author drew heavily on personal experience as a father rather than a professional.

This incident was profoundly upsetting and revealed a learning need, namely how best to support a suddenly bereaved child. Seeking answers, a personal reflection was undertaken, as this clinician finds the acquisition of knowledge following traumatic emergency calls reassuring and comforting if it leads to improved clinical practice.

## Method of literature search

Using the OneSearch digital search tool, academic papers were sought. Boolean operators, key words/phrases searched included: bereave* child*; child* traumatic grief; parent* death AND prehospital OR pre-hospital OR ambulance OR Paramedic OR EMS. Literature was selected related to child grief/bereavement and sudden/unexpected deaths, with ‘child’ being defined as subjects up to and including age 18.

## Traumatic grief in children

Childhood traumatic grief occurs when normal adaptive grief is overwhelmed by symptoms of trauma (Cohen and Mannarino, 2011). Bereavement-related post-traumatic stress disorder and prolonged grief disorder are recognised sub-categories, the former characterised by intrusive thoughts/images associated with death (Stoppelbein and Greening, 2000), the latter manifesting as persistent distress, shock, disbelief and altered worldviews (Boelen and Smid, 2017).

The cognitive processes needed to understand death necessitate comprehending inalienable truths: all humans die (inevitability), all living entities die (universality), death is permanent (irreversibility), all physical and psychological functions end on death (cessation) and death results from bodily process breakdown (causality) (Speece & Brent, 1984). The inevitability and irreversibility of death can be comprehensible to children as young as 5, yet universality and cessation are not understood until after age 6 and causality until age 10 (Panagiotaki et al., 2018). A linear developmental understanding of death complicates understanding, contributing to maladaptive cognitions such as self-blame (Cohen and Mannarino, 2011).

## Impact of childhood bereavement

Bereaved children commonly experience intense fear, helplessness or horror, which can lead to traumatic grief. Studies detail vulnerabilities, difficulties and undesirable impacts on health, with psychopathology and somatic issues commonly reported (Griese et al., 2018). There is increased risk of mental health/behavioural disorders, including anxiety/depression, aggression and academic and relationship problems (Christ, 2010). Social withdrawal/attachment issues, suicide, substance use, risky behaviours, social difficulties and poorer coping skills persist as long-term risks (Høeg et al., 2018).

## Death comprehension in children

It is important to consider the perspective and developmental cognition of BC as they journey through adolescence and adulthood. Despite limited studies, there are certain commonly agreed upon actions which are likely to help or hinder BC (Weber et al., 2019).

Age-related processing of parental death (PD) based on the work of Dowdney (2008), Panagiotaki et al. (2018), Aynsley-Green (2017) and Child Bereavement UK (2018) are detailed below. Knowledge of childhood developmental stages can assist clinicians communicating with BC who need information given in age-appropriate language. Children are very literal, so euphemisms should be avoided.

Berg et al. (2016) suggest that younger children are at greater risk of adverse outcomes and may erroneously attribute PD to their actions/inactions (Child Bereavement UK, 2018). Younger children engage in egocentric thinking, having difficulty grasping alternative perspectives (Biank and Werner-Lin, 2011); consequently, ‘magical thinking’, confusion, guilt, regret and self-blame are harmful reactions sometimes seen following PD (Christ, 2010). Younger children may fixate on worries that adults do not consider, such as concern about the body getting cold or lonely or that they may have caused the death (Sudden, 2014).

As understanding increases, questions regarding death follow. Repeated explanations are necessary as young children struggle to grasp the permanence of death, often expecting the deceased to return. Beliefs that thoughts and feelings cause/reverse death commonly continue until around age 7.

As children get older, paradoxical beliefs are common, and explanations concerning death reveal coexisting contradictory thoughts about biological and meta-physical/spiritual ideas (Panagiotaki et al., 2018).

Primary school aged children begin to comprehend the permanence and universality of death, but with limited understanding. This age group may develop heightened fears that others important to them will die. They frequently have questions about their parent’s death, exhibit somatic symptoms of grief such as disturbed sleep and intensification of emotions and can struggle to express or understand overwhelming feelings.

Secondary school aged children increasingly realise how PD has impacted their life, identity and future. Often feeling isolated or different from their peers, intense grief can re-emerge suddenly. Changeable feelings, becoming withdrawn and exhibiting increased risk-taking behaviours are also reported.

Reinterpreting the life and death of a parent occurs at successive developmental stages as cognition advances and emotions mature. These transitions should not be interpreted as instability; rather, they are normative restructuring (Carter and McGoldrick, 1999).

## Results

Children should be allowed to ask questions and know that this is ok (Aynsley-Green, 2017). Children’s capacity to deal with harsh truths is frequently underestimated by adults. Information voids created by misguided attempts to shield children become filled with fantasies likely to be more distressing. Adults often have reservations about children viewing bodies, yet research suggests that when prepared and not forced, children usually find it beneficial, although the timing of this would seem to depend upon the child and environment and ought to be judged on a case-by-case basis (Child Bereavement UK, 2018).

A significant influence on the development of childhood traumatic grief is the surviving parent/carer’s ability to engage and support BC alongside their own loss and changing circumstances. Encouragingly, supported surviving parents typically adopt and develop protective behaviours which benefit their children (Haine et al., 2006). Emotionally available parents who foster BC’s continuing bond with the deceased and promote open, honest communication and expression of feelings are helpful for BC (Karydi, 2018).

Children periodically re-evaluate PD, which can result in renewed traumatisation and/or attachment of new meaning or lived experience (Aynsley-Green, 2017). Children may do this because they are unable to fully resolve bereavement issues until the cognitive and emotional maturity needed to understand PD occurs (Biank and Werner-Lin, 2011). This theory is controversial, though, as other studies have found that most BC are resilient and follow normal developmental trajectories (Boelen et al., 2017).

Despite the paucity of evidence-based approaches to care provision for pre-hospital BC, clinicians could prove helpful. Fredman (1997) found that healthcare professionals frequently reported feeling unqualified to discuss death with children, believing it to be the domain of specialist trained experts only. This often translates into inaction and reluctance to address the needs of children for fear of saying or doing the wrong thing. Fredman concluded that carers should utilise personal experiences as helpful resources instead of deferring to professional theories.

## Management

Support for BC, though needed, is often lacking (Weber et al., 2019). The quality of care offered to children following bereavement can greatly impact future well-being (Nickerson et al., 2013), and even relatively brief supportive interventions can mitigate future debilitation. What exactly quality care should be remains uncertain, and further research is needed.

Worden (1996) offers guiding principles for supporting BC, urging clinicians to give clear, comprehensive information, address fears and anxieties and provide reassurance that the BC are without blame. Children appreciate being listened to carefully, and benefit from having their feelings validated, being included in discussions and being comforted when overwhelmed (Christ, 2010). Child Bereavement UK (2018) advise that BC’s questions should be answered honestly in simple age-appropriate language, with every effort made to remove uncertainties/confusion.

Referral to treatments such as grief and trauma intervention which blend cognitive behavioural therapy and narrative practices can be helpful, encouraging children to explore restorative stories about what has happened and thereby promoting future well-being and development while diminishing harmful stimuluses (Salloum, 2015).

Clinicians interacting with people who have experienced parental bereavement need to be cognisant that childhood development means welfare screening and interactions ought to be regularly considered, particularly around landmark occasions and key stages of development such as house moves, changes of school, puberty, graduation, marriage, childbirth and divorce. Such events or biological developments often herald re-evaluation, reinterpretation and/or potential renewal of grief trauma / adverse behaviours over the course of the BC’s lifetime (Biank and Werner-Lin, 2011), although prevalence and susceptibility remain unclear.

## Limitations

Further relevant studies may have been unearthed using additional search tools and strategies. The support and resources offered by charity organisations have not been discussed or explored.

## Conclusion

More research and initiatives are needed to support BC in the immediacy of traumatic life-changing parental loss. The current absence of clinician training and organisational support mechanisms in pre-hospital environments is lamentable. Ambulance services are well placed to provide comfort, care, signposting and guidance to this vulnerable group when a PD occurs in the community, the benefits of which are unmeasured. Relatively modest training on childhood bereavement, coupled with greater interaction with bereavement charities/organisations, might reasonably be supposed to have substantial positive impacts on children and surviving parents. Such endeavours may also improve the well-being of clinicians who manage these incidents, but further exploration is needed.

Improved awareness and training will likely benefit BC in the immediacy of tragedy and hopefully provide a stable platform that can be built on to support them onwards throughout their lives (Høeg et al., 2018).

Clinicians ought to be reassured that thoughtful, compassionate, honest and open engagement tailored to BC’s needs is highly likely to provide comfort and very unlikely to do harm. Moreover, clinicians should resist ‘being seduced by the empowerment of specialist death knowledges – those professional theories and approaches that tell us what should happen and what is the correct way to respond’ (Fredman, 1997, p. 13). Instead, healthcare professionals may hope to trust that sensitive, thoughtful interactions are better than repressed interactions and could possibly limit the development of harmful psychopathologies and muted happiness in these vulnerable children.

## Guarantor statement

BM acts as the guarantor for this article.

## Conflict of interest

None declared.

## Funding

None.

## Informed consent

Consent was gained from next of kin.
